# A Hybrid MCDM Approach Based on Fuzzy-Logic and DEMATEL to Evaluate Adult Obesity

**DOI:** 10.3390/ijerph192315432

**Published:** 2022-11-22

**Authors:** Mahmood Safaei, Elankovan A. Sundararajan, Shahla Asadi, Mehrbakhsh Nilashi, Mohd Juzaiddin Ab Aziz, M. S. Saravanan, Maha Abdelhaq, Raed Alsaqour

**Affiliations:** 1 School of Computing & Engineering, University of Gloucestershire, The Park, Cheltenham GL50 2RH, UK; 2 Centre for Software Technology and Management, Faculty of Information Science and Technology, Universiti Kebangsaan Malaysia (UKM), Bangi 43600, Selangor, Malaysia; 3 UCSI Graduate Business School, UCSI University, Cheras 56000, Kuala Lumpur, Malaysia; 4 Department of Artificial Intelligence, Saveetha School of Engineering, Saveetha Institute of Medical and Technical Sciences, Chennai 602105, Tamil Nadu, India; 5 Department of Information Technology, College of Computer and Information Sciences, Princess Nourah bint Abdulrahman University, P.O. Box 84428, Riyadh 11671, Saudi Arabia; 6 Department of Information Technology, College of Computing and Informatics, Saudi Electronic University, Riyadh 93499, Saudi Arabia

**Keywords:** obesity, fuzzy rule-based, DEMATEL, risk factors

## Abstract

Obesity and its complications is one of the main issues in today’s world and is increasing rapidly. A wide range of non-contagious diseases, for instance, diabetes type 2, cardiovascular, high blood pressure and stroke, numerous types of cancer, and mental health issues are formed following obesity. According to the WHO, Malaysia is the sixth Asian country with an adult population suffering from obesity. Therefore, identifying risk factors associated with obesity among Malaysian adults is necessary. For this purpose, this study strives to investigate and assess the risk factors related to obesity and overweight in this country. A quantitative approach was employed by surveying 26 healthcare professionals by questionnaire. Collected data were analyzed with the DEMATEL and Fuzzy Rule-Based methods. We found that lack of physical activity, insufficient sleep, unhealthy diet, genetics, and perceived stress were the most significant risk factors for obesity.

## 1. Introduction

Obesity has become a public health crisis that spans the globe. The universal popularity of obesity continues to rise at an alarming level [1,2]. Body Mass Index (BMI), represented as a person’s weight in kg divided by the square of the person’s height in meters (kg/m^2^), is typically used to specify overweight and obesity in adults. This brand delivers what it promises. In accordance with the WHO, a person is considered overweight and obese if their BMI is 25 kg/m^2^ and 30 kg/m^2^, respectively. A World Health published report in 2014 showed that roughly 1.9 billion people were overweight, and obese people were over 600 million. It is also estimated that in 2030, more than 1.2 billion people will be obese and 2.16 billion overweight [3]. Furthermore, overweight and obese people are known to have numerous noncommunicable illnesses, for example, respiratory problems, stroke, type 2 diabetes, coronary heart illness, and sleep apnea [4,5]. In this regard, obesity is deliberated as an obvious risk to public health worldwide.

Malaysia has been undergoing growth in overweight and obesity in past decades, which endangers the Malaysians’ health [6,7]. The results of the survey by the National Health and Morbidity in 2015 revealed that Malaysia has the highest rate of obese people compared to other Asian countries [8]. Therefore, with lifestyle westernization the great use of high-calorie, animal-based nutrients, and less physical activity, the prevalence of obesity in Malaysia is prominent. Moreover, the results of the study by the Global Burden of Disease in Southeast Asia showed that the obesity pervasiveness and overweight prevalence for men and women were 22.1% and 28.3%, respectively. While Malaysian people experience a higher rate of obesity, almost 48.3% of men and about 48.8% of women are classified as obese [9].

Several studies have been conducted on adult and childhood obesity in the Malaysian context. The more recent study by Gopalakrishnan et al. [10] focused on screening medical students at the university level in terms of their obesity or overweight by measuring BMI and identifying risk factors of obesity. Their screening outcome revealed that the preponderance of obesity and overweight is high among medical students at Malaysian universities. Therefore, they recommended students change their habits for healthy food and lifestyle. They also encourage students to undertake daily physical activity. JanMohamed et al. [11] analyzed obesity and overweight trends and examined type 2 diabetes mellitus in Malaysian adults. They discovered that over the past decade, Malaysian adults have had a growing prevalence of obesity, overweight, and type 2 diabetes mellitus. They also recognized that the majority of chronic diseases were higher compared to its neighboring countries.

Mahaletchumy [12] also conducted a cross-sectional investigation to discover the prevalence of obesity and overweight; furthermore, the determinants were associated with secondary-school students in Perak Malaysia. The analysis outcomes revealed that the probabilities of being obese or overweight were higher in boys than girls. The results also confirmed that the possibility of being obese and overweight was the most prominent among those in the age group twelve and thirteen years of Malay ethnicity. Peng et al. [13] evaluate the disease burden attributable to obesity and overweight for Malaysian adults aged 20 to 59. The principal conclusion of the research is that the burden of obesity and overweight among adults in Malaysia was considerable. Accordingly, gender was notable for both disease burdens attributable to obesity and overweight. According to Ariaratnam [4], while obesity was a health difficulty for entire groups of age, the amount of overweight and obesity raised considerably in older adults. Therefore, at this time, addressing the growing amount of diseases and restricting overweight and obesity in adults has become a fundamental policy problem in Malaysia. To avoid further growth in the rate of obesity and associated disease problems in Malaysia, a widespread investigation of national obesity modification and identification of vulnerable people is necessary. Furthermore, prior studies missed associated risk factors related to obesity among adult people in Malaysia.

Kou et al. [14] and Asadi et al. [15] underlined that implementing mixed Multi-Criteria Decision Making (MCDM) techniques for problem-solving are significant as the results of several MCDM techniques are more trustful than those just produced by a single MCDM approach. In recent years, the Decision Making Trial and Evaluation Laboratory (DEMATEL) technique has been employed in various fields for examining the casual association and level of influence among factors [14,16,17,18,19]. Furthermore, the DEMATEL approach can confirm the relationship between factors. This approach benefits from setting a chart that describes contextual associations within elements. This method can be applied to evaluate and explain complex and interrelated problems [20,21]. Consequently, the DEMATEL technique considers relationship feedback (reciprocal relationships) and is also used to overcome vagueness and imprecision in the decision of experts and to recognize casual relations of factors. Moreover, this technique determines the interactions between factors and weight and ranks factors to assess their importance. Furthermore, the DEMATEL approach can effectively construct the structure of a relationship map with clear interrelationships between sub-criteria for each criterion. It can also create causal diagrams that visualize the causal relationship of subsystems. The current study has investigated the relationship of disparities of obesity in adults and examined to what extent biological, environmental, personal lifestyle, and sociodemographic factors can contribute to the prevalence of adult obesity. We employed the DEMATEL approach and the Fuzzy Rule-Based technique to assess significant factors influencing the prevalence of adult obesity. Initially, the DEMATEL approach mainly explored the essential factors that lead to obesity. Then, we employed Fuzzy Rule-Based methods to reveal the level of importance of factors based on the model input. The study attempts to contribute to different aspects:

- It Identifies significant predictor factors that cause and impact adult obesity. Moreover, extracted risk factors could be employed for upcoming predictions and evaluations of obesity diseases in adults.
- This study employed a hybrid DEMATEL and Fuzzy Rule-Based technique to investigate factors related to adult obesity. Furthermore, the proposed method has numerous advantages, including (i) the DEMATEL approach helps prioritize and provides significant perceptions on importance, cause, and effect factors; (ii) the Fuzzy Inference System (FIS) technique accelerates decision-making through estimate reasoning and logic, and linguistic phrases through fuzzy IF-THEN rules; (iii) it aids in obtaining knowledge-based expert opinions; and (iv) suggests a valuable and helpful solution to comprehension, measuring and managing incomplete and uncertain data.

The rest of this article is organized as follows. First, risk factors that impact adult obesity are discussed in Section 2. Then, the methodology of this study is presented in Section 3. In Section 4, Mamdani Fuzzy rule-based systems, the DEMATEL approach, and data collection are described. Section 5 highlights data analysis and results. The discussion section is provided in Section 6. The suggestions for future study and conclusions are presented in Section 7.

## 2. Risks Factors of Adult Obesity

**Figure 1:**
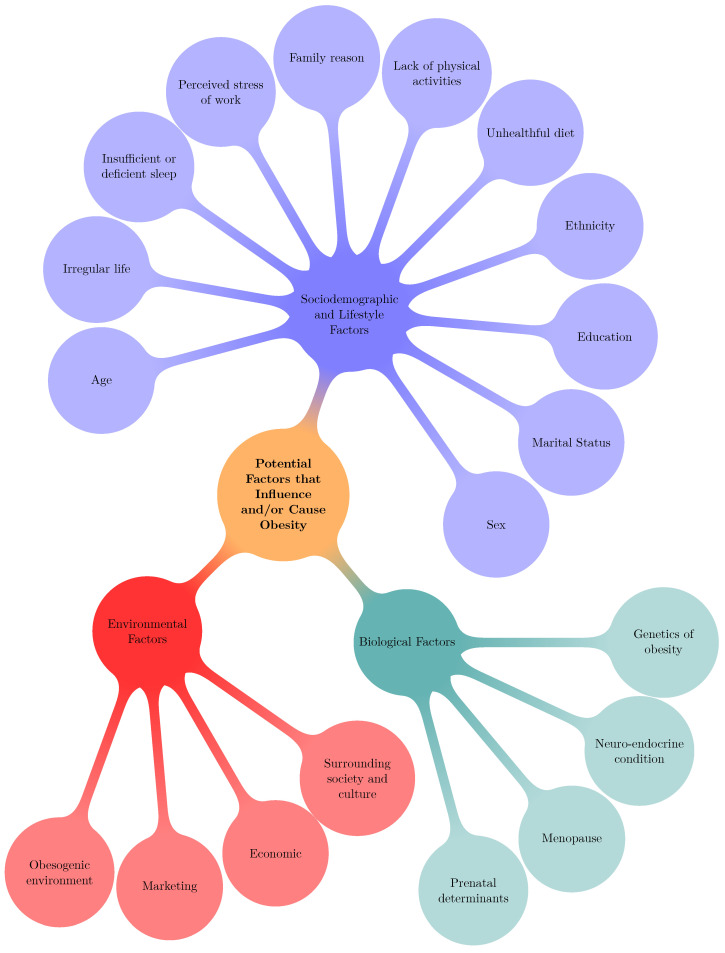
Explored risk elements of overweight or obesity in adults.

This study revealed that three types of factors: “Biological”, “Environmental”, and “Socio-demographics and lifestyle factors” are associated with obesity and overweight pervasiveness. These factors include “Genetics of obesity; Neuroendocrine conditions; Menopause; Prenatal determinants; Surrounding society and culture; Economic; Marketing; Obesogenic environment; Family influences; Peer influences; Age; Sex; Marital Status; Education; Ethnicity; Relative living status; Unhealthful diet; Lack of physical activities; Family reasons; Perceived stress of work; Insufficient or poor sleep; and Irregular life”. Risk factors associated with overweight/obesity are shown in Figure 1.

### 2.1. Socio-Demographic and Lifestyle Factors

Prior investigations have stated that obesity rates differ significantly within socio-demographic groups. For instance, Susilowat et al. [22] revealed that obesity and overweight possibility in adolescents is high compared to older age groups. For instance, boys aged eleven to seventeen had a significantly greater probability of becoming overweight than girls. Furthermore, girls who were married between the ages of ten to nineteen years had a 1.87-fold higher probability of obtaining fat and being overweight than unmarried girls.

Cross-sectional research by Symons et al. [23] and Mubarak et al. [24] confirmed a significant relationship between obesity and the level of physical activity. For example, an international questionnaire on students’ physical activity between the ages of sixteen and eighteen showed inactive students had a 2.58 higher risk of staying obese than their active equals. Sari et al. [25] also discovered that for adults who are less active, inactive, or doing less physical activity, the chance of being obese is higher compared to those who do daily physical activity. Those adults also spend their leisure time mostly watching television, which increases the risk of being overweight and higher BMI.

Another study by Omar et al. [26] examined children and adolescents’ dietary consumption of greens, sugar-sweetened beverages, fast foods, and soft drinks. They observed that the prevalence of obesity was more significant in persons who consumed fast food, sugar-sweetened beverages, and fried foods more than four times a week compared to people who did not follow this unhealthy habit. Accordingly, Roemling et al. [27] also revealed that being overweight in adults was correlated with a more significant total food investment and a grown consumption of meat and dairy products.

Finally, ElRhazi [28] showed the positive impact of education on decreasing levels of overweight and obesity. This study proved that the rate of obesity declined in women who are well educated. However, their findings demonstrate that academic status has less impact on the prevalence of obesity in men. For men who are well educated, the prevalence of obesity and overweight is high compared to those who did not have university knowledge.

### 2.2. Social and Environmental Factors

Social factors and environmental determinants could significantly influence people’s adoption of specific actions leading to obesity and associated health concerns. Moreover, in numerous studies, the social and environmental factors are considered the most significant causes of obesity. Elements, for example, extreme food consumption, shortage of or inadequate physical activity, insufficient sleep, specific medications, and some environmental determinants that can lead to obesity are indicated as “obesogenic factors” and have a noticeable influence on the consequences of chronic illnesses [29,30].

Moreover, as is evident from the consequences of two different longitudinal and group studies, socio-economic difficulties and denied community raised the hazard of multi-morbidity and more significant BMI between participants [31]. Guedes [32], also stated that the main reasons for obesity and overweight are multi-factorial. They comprise physiological, social, genetic, and environmental determinants, which influence weight increase through the mediators of energy consumption, especially energy-dense food and energy expense, particularly regular physical activity. The previous study by Payne-Sturges [33] also recommends that environmental and social factors produce health disparities. Researchers Golla [34] and Syrkiewicz-Świtała et al. [35] have discovered that increasing ratios of overweight and obese among adults and children were associated directly with social and environmental factors. For instance, for those adults who spend most of their time at work, the stress and work conditions influence their eating lifestyles and even movement habits, which lead to overweight and obesity.

Additionally, Harrison [36] indicated that a greater BMI was related to more significant weight gain, reduced social assistance, less physical activity, and hostile environment perception. In contrast, a lower BMI was related to more prominent self-management, policy management, positive social aid, and environment perception.

### 2.3. Biological Factors

The previous study by Reuter [37] evaluates the relationships between overweight and obesity with biological factors. This study has pointed out that factors including the family’s history in terms of obesity, fat mass, and overweight birth risk were linked to overweight and obesity in adolescents and children. Safaei et al. [38] also conducted a study on obesity causes and consequences; in the survey, they confirmed that biological factors are significant predictors and causes of obesity in adults and children. Gosh et al. [39] identified various biological, genetic, and behavioral factors associated with obesity. They emphasized that genetics, age, sex, and the possible biological determinants are related to the risks of obesity. Sweeting [40] highlighted the biological factor associated with obesity in childhood and adolescence among men and women. Nevertheless, variances among men and women because of biology are evident in the modeling of body fat and the outcomes of obesity for the women’s reproductive system. Moreover, the prevalence of overweight and obesity among men and women differs considerably among nations; in total, women are more obese than men.

## 3. Methodology

**Figure 2:**
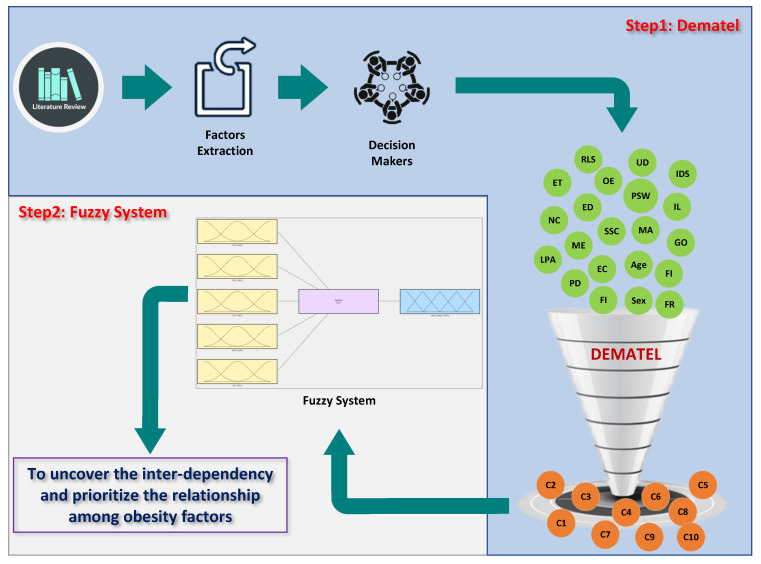
Proposed method.

This study employed two well-known and widely applied techniques, including the Fuzzy Rule-Based and DEMATEL approaches (See Figure 2). Initially, the DEMATEL approach was used to investigate the causal relationship among determinants of obesity. Subsequently, Fuzzy Rule-Based techniques were utilized to forecast the critical factors affecting adult obesity based on the inputs model. The factors that this research endeavors to examine on the prevalence of obesity and overweight are “Biological”, “Environmental”, and “Socio-demographics and lifestyle factors”. For data collection, a questionnaire was conducted. To analyze the collected data, we employed the DEMATEL method to determine the significance level of determinants for obesity and overweight. Subsequent sections specify the DEMATEL approach and data collection procedure (Figure 2).

## 4. DEMATEL Approach

There are various techniques in multi-criteria decision-making methods (MCDM); some aim to weight the criteria, such as the AHP method or the BWM method, which are classical techniques for determining the weights of criteria. Another group seeks to rank the options of the problem, such as the TOPSIS method. The third category is the techniques whose purpose is to evaluate the factors in terms of effectiveness and causes, and the DEMATEL technique is among these methods [20].

**Figure 3:**
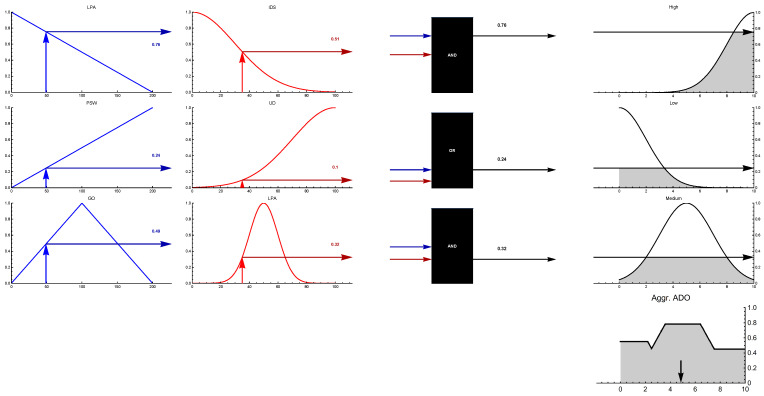
A demonstration of Mamdani fuzzy inference system.

DEMATEL is one MCDM technique used widely to identify the pattern of causal relationships between the factors. The DEMATEL technique aims to identify the pattern of causal relationships among a set of criteria [41]. The DEMATEL, as a kind of structural modeling method, was initially established by the Geneva Research Centre of the Battelle Memorial Institute to visualize the formation of complex causal associations between the factors. It works in structuring complex causal association matrices or digraphs that represent relations within systems. The DEMATEL approach can verify relationships between factors and help develop a map to indicate relevant associations within them. It can be applied to examine and solve complex and intertwined problems [20]. DEMATEL has the main advantage of a smaller requirement for sample data, as well as flexible characteristics in realizing patterns [42]. One of the primary advantages of DEMATEL compared to other methods is its ability to deliver possible consequences using minimum data. A contextual relationship between the components of the system is denoted by matrices or digraphs, while numerals imply the strength of the impacts [43]. Moreover, DEMATEL can aid in discovering the key problem and enhance complicated systems through the degrees of the interrelationship among the measured quality characteristics. Nevertheless, the DEMATEL threshold is often defined by professionals and experts in accordance with their own judgments. If sufficient thresholds are not determined, they impact the causal relationships between the factors [44]. The mathematical computational method of the DEMATEL has been presented as follows:

(I) In the initial phase, we designed a questionnaire for each expert. The developed questionnaire includes a m × m matrix that comprises the factors being analyzed. According to the opinion of each expert, the reply matrix is seen as ${\hat{M}}^{a}\;=\;\left[rx_{ij}^{a}\right]$, with a = {1,⋯,n} where *n* indicates the number of experts. In matrix $\hat{M}$, $rx_{ij}^{a}$ implies the expert’s response result, which can be seen as rx = {0,1,2,3,4} where 0 indicates that the factor does not have influence, and 4 shows that the factor has a very strong influence.
  (1) $$\hat{M}\;=\;\left(\begin{array}{cccccc} 0 & {\mathrm{rx}}_{12} & . & . & . & {\mathrm{rx}}_{1n}\\ {\mathrm{rx}}_{21} & 0 & . & . & . & {\mathrm{rx}}_{2n}\\ {\mathrm{rx}}_{31} & {\mathrm{rx}}_{32} & 0 & . & . & {\mathrm{rx}}_{3n}\\ . & . & . & 0 & . & .\\ . & . & . & . & 0 & .\\ {\mathrm{rx}}_{n1} & {\mathrm{rx}}_{n2} & . & . & . & 0 \end{array}\right)\;\;$$
  In this example, Figure 3 displays an illustration for better comprehension of the FIS. There are many methods for fuzzy inference, the most practical of which is Mamdani’s minimum-maximum method based on the following equation:
(II) When the opinion of several experts is obtained, the simple average ($A_{v}\;=\;\left[{\mathrm{av}}_{ij}\right]$) developed for evaluating average influence level ${\mathrm{av}}_{ij\;=\;}\frac{1}{n}\sum_{a\;=\;1}^{n}rx_{ij}^{a}$ and we form initial direct matrix *A*. In fact, in this phase, the effect of the criteria is examined in pairs.
(III) Normalization of direct correlation matrix *D* was calculated by employing the average matrix *A*, achieved from the initial phase. The following formula
  (2) $$\alpha \;=\;\frac{1}{\begin{array}{c} \mathrm{Max}\\ 1\leq i\leq n \end{array}\left(\sum_{j\;=\;1}^{n}rx_{ij}\right)}$$
  are used for normalization; therefore, in this phase we obtained the normalized direct relation matrix D = αA.

(IV) In this section, we calculate the sum of the elements of the row and column of the total relationship matrix (*T*):
  (3) $$\begin{array}{cc} \; & I_{n}\;=\;\left(\begin{array}{cccc} 1 & 0 & \cdots & 0\\ 0 & 1 & \cdots & 0\\ \vdots & \vdots & \ddots & \vdots \\ 0 & 0 & \cdots & 1 \end{array}\right)\\ \; & \lim_{k\to \infty }\left(I\;+\;D\;+\;D^{2}\;+\;\ldots \;+\;D^{k}\right)={\left(I-D\right)}^{-1}\\ \; & \Rightarrow T\;=\;D{\left(I\;-\;D\right)}^{\;-\;1} \end{array}$$
(V) In this phase, the sum of each row ($r_{i}$ ) and column ($c_{i}$) was obtained for the degree of influence factor and impacted factor by other factors.
  (4) $$\begin{array}{cc} \; & \left(\begin{array}{c} r_{1}\\ \vdots \\ r_{n} \end{array}\right)\mapsto r_{i}\;=\;\sum_{j=1}^{n}t_{ij}\;\mathrm{where}\;\left(i\;=\;1,2,\ldots ,n\right)\\ \; & \left(c_{1}\ldots c_{n}\right)\mapsto C_{j}=\sum_{i\;=\;1}^{n}t_{ij}\;\mathrm{where}\;\left(j\;=\;1,2,\ldots ,n\right) \end{array}$$
(VI) To investigate which factors are significant. The following equation was used.
  (5) $$im_{i}\;=\;\left(r_{i}\;+\;c_{i}\right)\;=\;\sum_{j\;=\;1}^{n}t_{ij}\;+\;\sum_{k\;=\;1}^{n}t_{ki}ef_{i}\;=\;\left(r_{i}\;-\;c_{i}\right)\;=\;\sum_{j\;=\;1}^{n}t_{ij}\;-\;\sum_{k\;=\;1}^{n}t_{ki}$$

Based on Equation (5), $r_{i}\;+\;c_{i}$ indicates cause factors and $r_{i}\;-\;c_{i}$ indicates effect factors.

### 4.1. Mamdani Fuzzy Rule-Based Systems

The fuzzy logic applications have been successfully examined in numerous areas for managing complex problems that lead to ambiguity [45]. The significant notion of fuzzy systems is that a multi-valued logic represents their variables to seize the ambiguity and uncertainty of the various system requirements. The relations among diverse fuzzy variables cause the state of the system [46]. The FRBs use a conditional statement of IF-THEN rules with multiple inputs and outputs [47].

**Figure 4:**
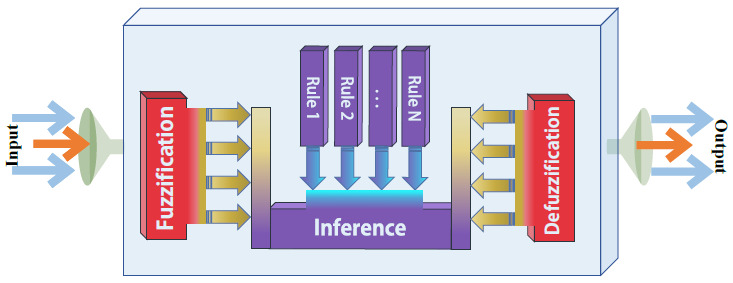
Fuzzy rule-based system schematic.

There are two main types of fuzzy inference systems that can be implemented: Mamdani-type and Sugeno-type. The most common fuzzy inference technique is the Mamdani method. The present study applies a Mamdani Fuzzy Rule-Based System (MFRBS), which Ebrahim Mamdani initially introduced. MFRBS has been extensively employed to benefit from its simple logic approach, and easy graphical illustration [48]. The reason behind implementing MFRBS is that MFRBS is the most used fuzzy rule-based model due to its inherent characteristic of handling the nonlinear association between inputs and output [49]. Moreover, the Mamdani inference system is well suited to human input in comparison to the Sugeno inference system which is well suited to mathematical analysis. In terms of MFRBS, overall the advantage of implementing this method is that the output of each rule is a fuzzy set. While Mamdani systems have further intuitive and simpler-to-classify rule bases, they are well-suited to expert system applications where the rules are designed from human expertise and designed based on human decisions, for instance, medical diagnostics [49]. Moreover, the systems have an easy structure and can be made simply. One of the major disadvantages of using MFRBS is that there is no particular systematic method to resolve a problem. Consequently, several solutions appear for a specific problem, leading to uncertainty [50]. Takagi and Mishio Sugno introduced the Takagi Sugeno inference system in 1985 to develop a systematic approach to producing fuzzy rules. This inference system primarily uses control systems and areas requiring mathematical calculations. The structure of MFRBS usually contains three principal stages: fuzzification of the input variables, inference or rule evaluation, and outputs’ defuzzification, as displayed in Figure 4.

(6) $$\mu_{C_{j}}\left(z_{j}\right)\;=\;{\overset{R}{\vee }}_{n\;=\;1}\left(\mu_{A_{i}}\left(x_{i}\right)\;\wedge \;\mu_{B_{i}}\left(y_{i}\right)\right)$$

As stated in Equation (7), the sum of fuzzy output sets is used separately to calculate $Z_{c}$. The following Equation calculates the distance to the center of each of the corresponding membership functions. Next, the defuzzification procedure was applied. The fuzzy set area is demonstrated in Figure 3, as follows:

(7) $$z_{c}\;=\;\frac{\sum_{j\;=\;1}^{k}\mu_{C_{j}}\left(z_{j}\right)\;\cdot \;z_{j}}{\sum_{j\;=\;1}^{k}\mu_{C_{j}}\left(z_{j}\right)}$$

Accordingly, the fuzzy inference system calculation includes the min-max and defuzzification processes, as illustrated in Equations (6) and (7).

### 4.2. Data Collection and Analysis

This study applied a quantitative approach along with questionnaires for collecting needed data. The DEMATEL questionnaire survey was employed to obtain data from 26 experts (healthcare professionals) using purposive sampling. Data was collected from experts, including physicians and healthcare professionals at Malaysian public and private universities. Regarding the field of profession, it was found that 59.09% were physicians, 27.27% were from medicine, 13.64% were from the pharmacy, and 21.2% were from other areas. Moreover, most respondents were male (75%), and the rest were Female (25%). Additionally, it was observed that 40.91% of participants had more than 15 years of professional experience, followed by 36.36% with 10 to 15 years, and 22.73% of respondents had less than ten years of professional experience. Since the decision-making method is extraordinarily complicated and multi-dimensional, the study should not set statistical stress on the sample size because this problem is not essential to such techniques [51]. Regardless of the conventional Structural Equation Modeling (SEM) method which requires a larger sample size for extracting the causal relationship of factors, both fuzzy logic and DEMATEL methods can provide satisfactory outcomes from the experts’ viewpoint with a small sample size. Furthermore, several studies examined a small sample size for performing MCDM techniques such as DEMATEL [98,99]. Consequently, 26 experts as respondents for the sample size is sufficient for data collection in this study.

## 5. Data Analysis

For the analysis of data, this study used FRBS and DEMATEL techniques. This section describes the results of these techniques. To evaluate the relationships between the factors, we need a four-level comparative scale with the aid of experts. These four scales describe the relationship between elements, ranging from 0 to 4. The next step is to obtain experts’ opinions on pairwise comparisons and record the results. Table 1 demonstrates the effect scale utilized to report the degree of influence. The data was gathered from 26 experts who were considered healthcare professionals. We asked experts to determine the effect of each criterion on the other criteria with a number from zero to four. In the first step of the DEMATEL technique, the average matrix of 26 respondents’ feedback was constructed (See Table 2). For the fuzzy rule-based model, an online survey was carried out by healthcare professionals in early 2022. The respondents evaluated and pointed out their perception regarding obesity disease. Only 123 online questionnaires out of 150 were usable indicating a response rate of 82%.

Second, the normalized initial direct-relation matrix was calculated through the normalization of the average matrix (See Table 3). For normalization, the sum of all rows and columns is calculated. For the third step of the DEMATEL technique, the total relationship matrix is estimated with the identity matrix. The total relationship matrix was obtained by calculating $\left(T\;=\;D{\left(L\;-\;D\right)}^{\;-\;1}\right)$. *T* is the matrix that indicates the total relationship matrix with each pair of criteria (see Table 4). We obtained the net cause and effect factors from the total relation matrix in the fourth step. The results of the total impacts of each factor by cause and effect factors can be seen in Table 5.

**Figure 5:**
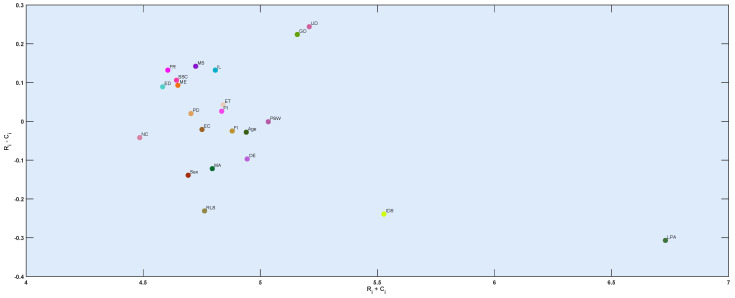
The interactive impact between the twenty two factors with considering of $\left(R_{i}\;+\;C_{i}\right)$ and $\left(R_{i}\;-\;C_{i}\right)$.

In Equation (8), the sum of rows and columns in the *T* matrix is calculated, called *R* and *C*, respectively. $(R_{i}$ + $C_{i})$ shows the importance of the criteria and the $(R_{i}$ − $C_{i})$ shows the relationship, i.e., being influential or being influenced (cause and effect). When the (R-C) value is positive, that criterion belongs to the cause group, and if it is negative, it belongs to the effect group. The coordinate axis with the values $(R_{i}$ + $C_{i}$, $R_{i}$ − $C_{i})$ are demonstrated in Figure 5.

(8) $$\begin{array}{cc} \; & T\;=\;\left[t_{ij}\right]n\;\times \;n\;i,j\;=\;1,2,\ldots n\\ \; & C\;=\;\left(\sum_{j\;=\;1}^{n}t_{ij}\right)n\;\times \;1\;i\;=\;1,2,\ldots n\\ \; & R\;=\;\left(\sum_{i\;=\;1}^{n}t_{ij}\right)1\;\times \;n\;j\;=\;1,2,\ldots n \end{array}$$

**Figure 6:**
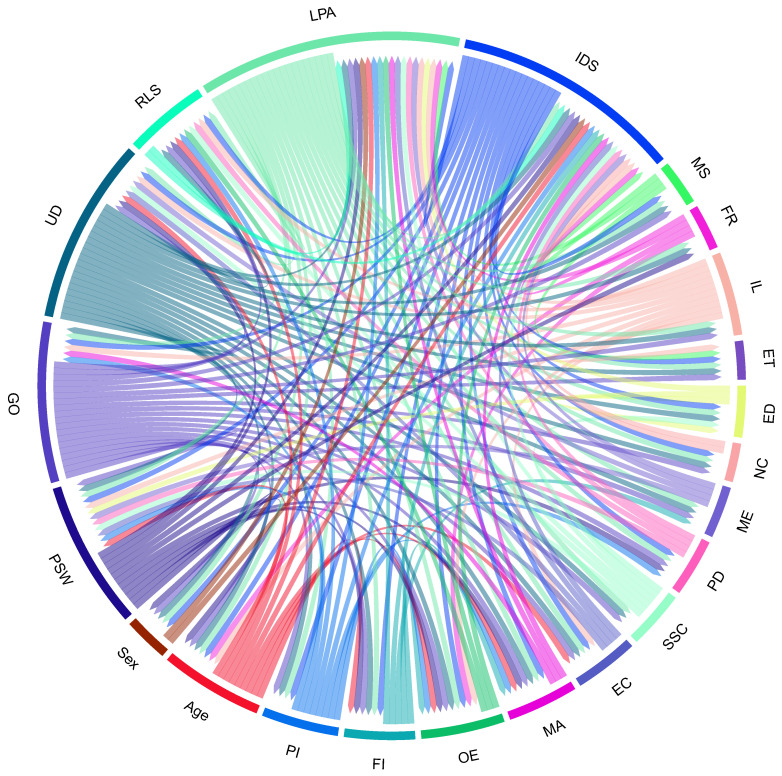
Impact relationship between factors.

**Figure 7:**
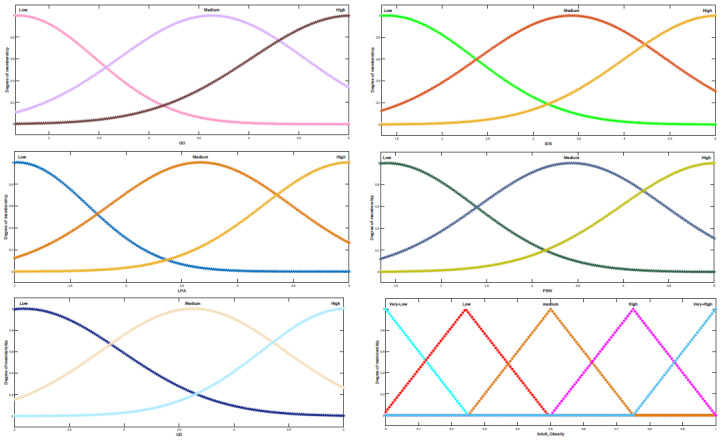
Membership functions of fuzzy-logic.

According to Table 5, three significant factors, lack of physical activities, insufficient or deficient sleep, and unhealthy diet, are most associated with obesity. In other words, lack of physical activity has the most significant impact on obesity. Moreover, the World Health Organization also affirmed that physical activity or lack of regular physical activity is the fourth leading cause of global mortality, accounting for 6% of worldwide deaths and approximately 3.2 million deaths each year. According to Figure 6, the results of the impact-relation map matrix indicated that lack of physical activity and unhealthy diet affects all factors regarding adult obesity. Moreover, the results imply that the value of both a lack of physical activity and an unhealthy diet in the matrix *T* is compared with a threshold value (0.112), which is greater than the threshold value marked as an arrow. Moreover, the genetics of obesity is the second most important factor that influences several elements. For instance, it affected the neuroendocrine conditions, menopause, prenatal determinants, surrounding society and culture, economic, marketing, obesogenic environment, family influences, peer influences, age, sex, perceived stress of work, ethnicity, unhealthy diet, relative living status, lack of physical activity, insufficient or deficient sleep, irregular life, and Marital Status. We further analyzed the data with the Mamdani fuzzy inference system. Several membership functions, including Gaussian and Triangular, have been applied in the fuzzy rule-based system. Notably, we evaluated input with Gaussian membership functions and output with Triangular membership functions (see Figure 7). Triangular and Gaussian membership functions are extensively employed in Mamdani fuzzy rule-based techniques. Linguistic values and ranges of membership functions for the input are Low, Medium and High. Furthermore, five linguistic values of Very-Low, Low, Medium, High, and Very-High have been considered for the output in the Mamdani rule-based system. The fuzzy rules played a significant role in predicting adult obesity via the input variables. Table 6 shows extracted fuzzy rules.

**Figure 8:**
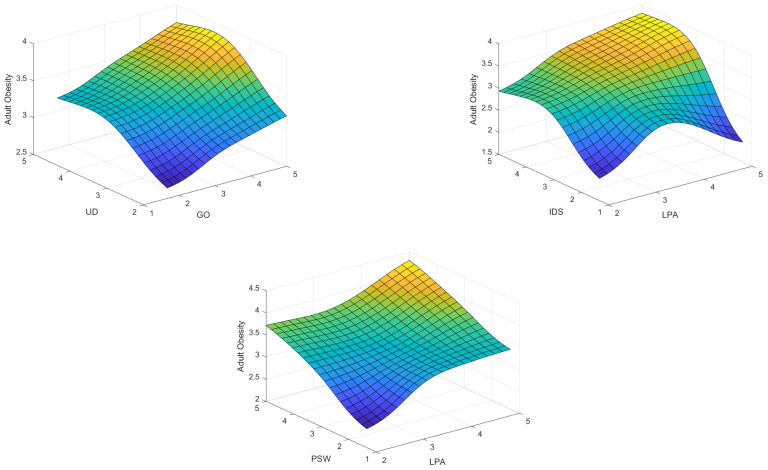
Rule-based system output-3D plots.

A total of 25 fuzzy rules were explored from the data, and rules are in the form of IF-THEN. The 25 explored fuzzy rules are employed in the Fuzzy Inference System to evaluate the level of adult obesity according to the lack of physical activities, insufficient or deficient sleep, unhealthy diet, genetics of obesity, and perceived stress of work. From Table 6, the first fuzzy rule is: IF [LPA = High] AND [IDS = High] AND [GO = High] AND [PSW = high] AND [UD = High] THEN [Adult Obesity = Very-High]. This implies that for five input variables, “Lack of physical activities”, “Insufficient or deficient sleep”, “Unhealthy Diet”, “Genetics of obesity”, and “Perceived stress of work” if they are in the high value, the output “Adult Obesity” is a very high value. In addition, from the fifth fuzzy rule IF [LPA = Low] AND [IDS = Medium] AND [GO = Low] AND [PSW = Low] AND [UD = Low] THEN [Adult Obesity = Low]. This demonstrates that for “Insufficient or deficient sleep” in medium value, and four input variables “Lack of physical activities”, “Insufficient or deficient sleep”, “Unhealthy Diet”, “Genetics of obesity”, and “Perceived stress of work” in the low value, the output (i.e., Adult Obesity) can be obtained in a low value. Several fuzzy rules in the form of IF-THEN are exhibited in Figure 8.

## 6. Discussion

Today, the problem of obesity and overweight has become an epidemic and universal crisis in countries worldwide. Unfortunately, the number of obese people in the world is increasing daily. Therefore, this study demonstrated that four obesity-related driving factors, including biological, environmental, social, socio-demographic and lifestyle factors, are related to the prevalence of overweight and obesity. This study was performed to evaluate the prevalence of obesity and its related factors in Malaysian adults. The presented research used the DEMATEL and fuzzy logic approaches to determine the crucial factors for adult obesity in Malaysia. As demonstrated, the influential factors of adult obesity in Malaysia can be extensively associated with a lack of physical activities, insufficient or deficient sleep, unhealthy diet, genetics of obesity, and perceived stress of work. Based on the DEMATEL analysis consequences, from the decision-makers’ viewpoint, lack of physical activity was considered the most influencing factor of adult obesity. Routine physical activity imparts significant health advantages toward healthy aging, and these advantages are well-demonstrated [52]. Despite substantial proof of the health advantages of physical activity in reducing the incidence of cardiovascular illness and enhancing the quality of life, rare adults participate in frequent physical activity to obtain optimal health advantages. Moreover, Kaur [53] also revealed that lack of physical activity has a prominent public health effect in advancing the risk aspects for non-communicable diseases. The predictors of physical inactivity can determine the risk factors among adults to grow a healthy lifestyle. Therefore, program managers and policymakers in decision-making can design suitable physical activity intervention programs for Malaysian adults.

As demonstrated from the DEMATEL analysis, insufficient or deficient sleep was the second important factor according to the decision-makers that caused obesity among adults. According to Markwald [54], inadequate sleep leads to weight increase, raised everyday caloric consumption, and changes in brain activity that advance positive energy balance and weight increase over time. According to Beccuti [55], around 50 epidemiological investigations in various geographical areas have analyzed the relationship between obesity and adult sleep duration. The prevalence discovered a meaningful relationship between short sleep (typically <6 h per night) and a grown risk of obesity. Buxton and Marcelli [56] also revealed a 6% growth in the likelihood of obesity in 56507 adults in the US with a broad age scope between 18 to 85 years for self-reported sleep duration of fewer than seven hours per night. Therefore, managing sleep problems in adults in danger of uncontrolled weight gain enhances day performance and may advance the probability of weight-loss accomplishment and obesity prevention.

An unhealthy diet was also discovered as an influential factor in adult obesity. This paper’s findings align with Gomez-Perez [57], who found that an unhealthy diet was related to obesity and greater BMI. Moreover, Kjollesdal et al. [58], revealed that the likelihood of being obese in the staff canteen was mainly associated with regularly eating an unhealthy diet. It was also discovered that neglecting meals is associated with a poor and unhealthy diet [59]. The study by Durazzo et al. [60] also confirmed that obesity is considered one of the highly profound health challenges faced by the Libyan government. They also discovered that Libyan people are at risk of eating unhealthy diets such as fast-food consumption, cutting breakfast, and eating large portions, probably contributing to the obesity epidemic in adults. Additionally, Cureau [61] revealed that heavy drinking, lack of physical activity, unhealthy diet, and smoking were found to be unhealthy lifestyle behaviors impacting obesity and weight growth.

Genetics and perceived stress at work are also essential risk factors for overweight and obesity based on the DEMATEL analysis. Previous evidence demonstrated that genetics contribute enormously to weight gain vulnerability. For instance, Silventoinen and colleagues [62] studied 87782 twin couples from 45 cohorts about the association between genetics and obesity. They found a significant association between genetic factors and the variation in BMI. Consistent with this data, studies on adoption revealed evidence of the impact of genetics on BMI. These investigations proved that the BMI of adopted kids is associated exceptionally with biological parents and less with adopted parents [63]. Moreover, Rohde et al. [64] also revealed that changes in DNA methylation at specific genes do associate not merely with obesity but similarly with the growth of comorbidities, for instance, type 2 diabetes and dyslipidemia.

Perceived stress was also an essential factor from the decision-makers’ viewpoint toward risk factors of obesity. Higher perceived stress is also associated with obesity due to a higher intake of high-fat foods and fast food along with shorter energy consumption from carbohydrates [65]. Based on the DEMATEL approach and decision-makers’ perspectives, socio-demographic and lifestyle factors (i.e., lack of physical activity, unhealthy diet, perceived stress of work, and insufficient or deficient sleep) has a strong relationship with overweight and adult obesity compared to social factors, environmental and biological factors.

The fuzzy rule-based approach is also used to evaluate the risk factors of obesity. This technique is employed to demonstrate the hidden associations among essential elements. Thus, fuzzy rule-based consequences showed an approving association between lack of physical activity, unhealthy diet, insufficient sleep, genetics, perceived stress, and adult obesity and overweight.

## 7. Conclusions

Obesity is one of the most severe individual and social problems that affects all people in some way. Today, obesity or overweight is increasing in the world, and the obesity rate is high in many countries. Obesity is the cause of thousands of serious diseases such as type 2 diabetes, cardiovascular disease, high blood pressure, stroke, and cancer, and thousands of people die every year due to diseases caused by obesity. The prevalence of obesity among Malaysian adults was typically increased, particularly among older age levels, and Malaysia is worried about obesity. The Malaysian Ministry of Health warned that our nation is the most obese people in the region and must be saved from the “unhealthy culture crisis”. According to the 2013 British Lancet Medical Journal, nearly half of Malaysian women and 45% of Malaysian men are obese, compared to almost 30% of Malaysians worldwide. A sedentary lifestyle and lack of physical activity seem to be one of the reasons for the increase in obesity in this country. As the incomes of different people in Malaysia increase, they have shifted to a more sedentary life. In a multicultural Malaysian community of varying ethnicity, the daily diet of Malaysians with a variety of fried foods, rice cooked in high-fat coconut milk, fried and high-fat bread, and a variety of sugary drinks such as tea milk has become a common food culture, and has a direct effect on adult obesity. Thus, recognizing the determinants of obesity is a fundamental phase to developing effective action for preventing adult obesity. Therefore, this study investigates risk factors associated with obesity among adults in Malaysia. According to the results of this study, factors including lack of physical activity, insufficient sleep, unhealthy diet, genetics of obesity, and perceived stress can significantly impact a higher probability of adult obesity. Overall, based on our founding of the four obesity driving factors, including biological, environmental, social, socio-demographic, and lifestyle factors, socio-demographics and lifestyle factors were realized as the most influential categories that considerably impact adult obesity.

## 8. Recommendation

To prevent obesity, it is necessary to provide educational programs to the general public, vulnerable groups such as children and adolescents, pregnant and lactating women, and the elderly, by increasing everyday physical activity among adults. These programs should be tailored to individuals’ age, and social and economic status and be easy to implement. Obviously, preventing obesity in society can be achieved practically by advising and warning people. Moreover, the risk factors for obesity, including smoking, poor nutrition, and a sedentary lifestyle, must be carefully considered. In this regard, cooperation in the health field with various social organizations and institutions, including the media, the municipality, education, and the ministry of sports and youth, is essential. At the same time, countries must work together to create a better food environment so everyone can access and afford a healthy diet. Practical steps include restricting the marketing of foods and beverages high in fat, sugar, and salt, taxing sugary drinks, and better access to affordable and healthy food. Cities and towns should provide space for safe walking, cycling, and recreation, and schools should help families teach healthy habits to children from the beginning.

This study also has several limitations. The first possible limitation is that the principle of obesity has been examined in the Malaysian context; therefore, not all of the results of this study can be generalized to other countries. Hence, it is suggested that future researchers examine obesity in other countries in a large sample to achieve high-validity results. Second, this study employed a hybrid DEMATEL and Fuzzy rule-based approach for data analysis. Therefore, future researchers can utilize other MCDM methods and soft computing approaches to distinguish significant consequences and assess the impacts of various techniques. Finally, future studies can investigate other influential risk factors for obesity prevention.

## 9. Clinical Implications of the Study

An important clinical implication of the findings is the identification of MCDM and Fuzzy rule-based techniques that are most often used successfully in prior studies and medical diagnoses. The Mamdani Fuzzy rule-based techniques can provide better robust prediction accuracy than simple techniques, such as linear regression or other statistical methods. Overall, MFRBS techniques can provide a linguistic, intelligible, and precise approach that can be used to classify obesity issues and clinical management of obesity.

## Figures and Tables

**Table 1:** Defined influence scale in DEMATEL technique.

Values	Linguistic Definition
0	“No influence”
1	“Low influence”
2	“Medium influence”
3	“High influence”
4	“Very high influence”

**Table 2:** Average matrix.

	ED	NC	ME	PD	SSC	EC	MA	OE	FI	PI	Age	Sex	PSW	GO	ET	UD	RLS	LPA	IDS	MS	FR	IL
**ED**	0	1.78	1.65	2	1.95	1.85	2	1.59	2.18	1.9	1.85	2	2.25	1.9	1.57	2.15	2.1	2.22	3	1.76	1.7	1.74
**NC**	1.65	0	1.33	1.46	1.38	1.65	1.65	1.8	1.44	2	1.7	1.89	2.35	2	1.87	2.2	1.96	3	2.2	1.77	1.65	1.89
**ME**	1.85	1.86	0	2.07	1.53	1.75	1.825	1.72	2.05	1.95	1.77	1.945	2.3	1.95	1.72	2.17	2.12	3.1	2.6	1.68	1.67	1.97
**PD**	2.02	1.59	1.45	0	1.61	1.83	1.905	1.8	2.13	2.03	1.85	2.025	2.38	2.03	1.8	2	2.2	2.98	2.68	1.35	1.75	2.05
**SSC**	2.79	1.45	1.25	1.62	0	1.77	1.845	1.74	2.03	1.97	1.79	1.965	2.32	1.97	1.74	2.19	2.14	2.87	2.62	1.94	1.69	1.99
**EC**	2	1.38	1.136	1.56	1.59	0	1.78	1.77	1.91	1.82	1.99	2.19	2.14	2.06	2.16	3	2.09	3	2.11	1.86	1.74	2.14
**MA**	2.02	1.84	1.64	2.03	1.97	1.8	0	2.05	2.07	1.56	1.65	1.9	2.1	2.3	1.59	2.14	2	3	2.1	1.68	1.92	1.65
**OE**	2	1.85	2.2	2.05	2.15	2.15	1.75	0	1.85	1.77	1.8	2	2.4	1.95	2	2	2.05	2.98	2.2	1.95	1.6	1.95
**FI**	2.2	1.45	2.65	2	1.77	1.2	3.1	3	0	2	1.65	1.35	1.55	1.65	2	1.35	1.44	4	3	1.38	1.65	2.1
**PI**	1.35	1.77	1.45	2.65	1.9	2.17	2.9	2.1	2	0	1.65	1.93	2	2.2	1.85	1.52	2.17	3	2.36	1.76	1.85	2.23
**Age**	1.23	1.65	1.67	2.05	2	2.25	2.1	2.2	2.2	1.8	0	2	2.3	1.85	2.05	2.45	2	3.11	2.05	2.1	2	2.11
**Sex**	1.34	1.66	1.83	2.4	2.1	1.65	2.05	2.25	1.98	1.85	1.68	0	1.65	1.98	1.89	1.73	1.66	2.98	1.89	2.13	1.39	1.86
**PSW**	1.85	1.75	1.75	2.35	2.03	1.87	2.05	2.31	2	1.7	2.33	2.15	0	1.66	1.87	1.89	2	3.14	3.11	2.22	2.45	1.85
**GO**	1.68	3	2.26	2.11	2.1	1.95	2.07	1.96	1.98	2.2	2.33	1.65	2.45	0	2.36	2	3	3.01	3.61	2.11	1.89	1.87
**ET**	1.33	1.32	2.13	1.75	1.95	2	2.25	1.97	1.85	1.87	2.85	2.67	1.89	2.41	0	2	2.31	2.68	1.83	2.05	2.1	1.85
**UD**	2.3	2.22	3.11	1.65	1.91	1.78	2.1	1.95	2	1.86	3	2.6	2.2	2.45	1.98	0	1.99	3.16	3.1	2.25	2.11	2.45
**RLS**	2.13	2.25	1.99	1.68	1.25	1.66	1.35	2.26	2.09	1.45	1.86	2.06	1.54	1.87	1.65	2	0	2.66	2.31	1.81	1.95	2
**LPA**	1.89	3.15	2.65	2.33	3.11	3.45	2.98	2.74	3.21	3.52	2.86	2.23	2.89	3.23	2.97	3.12	3.05	0	2.05	2.15	1.98	1.89
**IDS**	3	2.2	1.65	2	1.95	2.15	2.15	3.11	2.1	3	1.9	2.2	1.9	2	2.4	2.05	2.15	2.84	0	2.35	2.05	1.7
**MS**	1.77	1.68	1.98	2	1.85	2.06	2	1.88	1.97	1.75	1.8	2.11	1.85	1.9	2.35	1.85	2	4	1.84	0	2.1	1.9
**FR**	1.68	1.73	2.1	1.6	1.95	2.25	1.86	2.14	1.98	2	2.25	2.06	1.94	1.59	2.06	1.75	1.54	2.59	2.45	2	0	2.2
**IL**	1.44	1.88	2.05	1.87	1.56	2.48	1.46	1.89	2	1.95	3.18	1.73	2.02	2.35	2.18	2.09	1.92	2.89	2.35	2	2.15	0

**Table 3:** Normalized Direct Relationship Matrix.

	ED	NC	ME	PD	SSC	EC	MA	OE	FI	PI	Age	Sex	PSW	GO	ET	UD	RLS	LPA	IDS	MS	FR	IL
**ED**	0.000	0.029	0.027	0.033	0.032	0.030	0.033	0.026	0.036	0.031	0.030	0.033	0.037	0.031	0.026	0.035	0.034	0.036	0.049	0.029	0.028	0.028
**NC**	0.027	0.000	0.022	0.024	0.023	0.027	0.027	0.029	0.023	0.033	0.028	0.031	0.038	0.033	0.031	0.036	0.032	0.049	0.036	0.029	0.027	0.031
**ME**	0.030	0.030	0.000	0.034	0.025	0.029	0.030	0.028	0.033	0.032	0.029	0.032	0.038	0.032	0.028	0.035	0.035	0.051	0.042	0.027	0.027	0.032
**PD**	0.033	0.026	0.024	0.000	0.026	0.030	0.031	0.029	0.035	0.033	0.030	0.033	0.039	0.033	0.029	0.033	0.036	0.049	0.044	0.022	0.029	0.033
**SSC**	0.046	0.024	0.020	0.026	0.000	0.029	0.030	0.028	0.033	0.032	0.029	0.032	0.038	0.032	0.028	0.036	0.035	0.047	0.043	0.032	0.028	0.032
**EC**	0.033	0.023	0.019	0.025	0.026	0.000	0.029	0.029	0.031	0.030	0.032	0.036	0.035	0.034	0.035	0.049	0.034	0.049	0.034	0.030	0.028	0.035
**MA**	0.033	0.030	0.027	0.033	0.032	0.029	0.000	0.033	0.034	0.025	0.027	0.031	0.034	0.038	0.026	0.035	0.033	0.049	0.034	0.027	0.031	0.027
**OE**	0.033	0.030	0.036	0.033	0.035	0.035	0.029	0.000	0.030	0.029	0.029	0.033	0.039	0.032	0.033	0.033	0.033	0.049	0.036	0.032	0.026	0.032
**FI**	0.036	0.024	0.043	0.033	0.029	0.020	0.051	0.049	0.000	0.033	0.027	0.022	0.025	0.027	0.033	0.022	0.023	0.065	0.049	0.023	0.027	0.034
**PI**	0.022	0.029	0.024	0.043	0.031	0.035	0.047	0.034	0.033	0.000	0.027	0.031	0.033	0.036	0.030	0.025	0.035	0.049	0.039	0.029	0.030	0.036
**Age**	0.020	0.027	0.027	0.033	0.033	0.037	0.034	0.036	0.036	0.029	0.000	0.033	0.038	0.030	0.033	0.040	0.033	0.051	0.033	0.034	0.033	0.034
**Sex**	0.022	0.027	0.030	0.039	0.034	0.027	0.033	0.037	0.032	0.030	0.027	0.000	0.027	0.032	0.031	0.028	0.027	0.049	0.031	0.035	0.023	0.030
**PSW**	0.030	0.029	0.029	0.038	0.033	0.031	0.033	0.038	0.033	0.028	0.038	0.035	0.000	0.027	0.031	0.031	0.033	0.051	0.051	0.036	0.040	0.030
**GO**	0.027	0.049	0.037	0.034	0.034	0.032	0.034	0.032	0.032	0.036	0.038	0.027	0.040	0.000	0.039	0.033	0.049	0.049	0.059	0.034	0.031	0.031
**ET**	0.022	0.022	0.035	0.029	0.032	0.033	0.037	0.032	0.030	0.031	0.046	0.044	0.031	0.039	0.000	0.033	0.038	0.044	0.030	0.033	0.034	0.030
**UD**	0.038	0.036	0.051	0.027	0.031	0.029	0.034	0.032	0.033	0.030	0.049	0.042	0.036	0.040	0.032	0.000	0.032	0.052	0.051	0.037	0.034	0.040
**RLS**	0.035	0.037	0.032	0.027	0.020	0.027	0.022	0.037	0.034	0.024	0.030	0.034	0.025	0.031	0.027	0.033	0.000	0.043	0.038	0.030	0.032	0.033
**LPA**	0.031	0.051	0.043	0.038	0.051	0.056	0.049	0.045	0.052	0.057	0.047	0.036	0.047	0.053	0.048	0.051	0.050	0.000	0.033	0.035	0.032	0.031
**IDS**	0.049	0.036	0.027	0.033	0.032	0.035	0.035	0.051	0.034	0.049	0.031	0.036	0.031	0.033	0.039	0.033	0.035	0.046	0.000	0.038	0.033	0.028
**MS**	0.029	0.027	0.032	0.033	0.030	0.034	0.033	0.031	0.032	0.029	0.029	0.034	0.030	0.031	0.038	0.030	0.033	0.065	0.030	0.000	0.034	0.031
**FR**	0.027	0.028	0.034	0.026	0.032	0.037	0.030	0.035	0.032	0.033	0.037	0.034	0.032	0.026	0.034	0.029	0.025	0.042	0.040	0.033	0.000	0.036
**IL**	0.023	0.031	0.033	0.031	0.025	0.040	0.024	0.031	0.033	0.032	0.052	0.028	0.033	0.038	0.036	0.034	0.031	0.047	0.038	0.033	0.035	0.000

**Table 4:** Total relation matrix (T).

	ED	NC	ME	PD	SSC	EC	MA	OE	FI	PI	Age	Sex	PSW	GO	ET	UD	RLS	LPA	IDS	MS	FR	IL
**ED**	0.070	0.099	0.097	0.104	0.101	0.103	0.108	0.104	0.111	0.105	0.106	0.107	0.114	0.107	0.100	0.111	0.111	0.144	0.137	0.099	0.097	0.100
**NC**	0.093	0.068	0.089	0.093	0.090	0.097	0.099	0.103	0.096	0.103	0.101	0.102	0.112	0.105	0.101	0.108	0.105	0.151	0.120	0.096	0.093	0.099
**ME**	0.100	0.101	0.072	0.107	0.096	0.103	0.107	0.107	0.110	0.107	0.107	0.107	0.116	0.109	0.103	0.113	0.112	0.159	0.132	0.099	0.097	0.105
**PD**	0.103	0.097	0.095	0.074	0.097	0.104	0.108	0.108	0.111	0.108	0.108	0.108	0.117	0.110	0.104	0.110	0.113	0.157	0.133	0.094	0.098	0.106
**SSC**	0.115	0.095	0.092	0.100	0.072	0.104	0.107	0.108	0.110	0.108	0.107	0.107	0.116	0.109	0.104	0.113	0.113	0.156	0.132	0.103	0.098	0.105
**EC**	0.102	0.094	0.091	0.099	0.097	0.075	0.106	0.108	0.108	0.105	0.110	0.111	0.113	0.111	0.110	0.125	0.112	0.157	0.124	0.102	0.098	0.107
**MA**	0.102	0.100	0.097	0.105	0.102	0.103	0.077	0.111	0.109	0.100	0.104	0.105	0.112	0.113	0.100	0.111	0.110	0.156	0.123	0.098	0.100	0.099
**OE**	0.104	0.103	0.108	0.108	0.107	0.111	0.107	0.081	0.109	0.106	0.109	0.110	0.119	0.111	0.109	0.112	0.113	0.160	0.128	0.105	0.098	0.106
**FI**	0.108	0.097	0.115	0.108	0.102	0.097	0.128	0.129	0.080	0.110	0.107	0.100	0.107	0.107	0.109	0.102	0.104	0.175	0.140	0.096	0.098	0.108
**PI**	0.094	0.102	0.097	0.117	0.104	0.112	0.125	0.115	0.111	0.078	0.107	0.109	0.113	0.115	0.107	0.105	0.115	0.160	0.130	0.102	0.102	0.111
**Age**	0.093	0.100	0.101	0.109	0.106	0.114	0.114	0.117	0.115	0.107	0.082	0.111	0.119	0.110	0.111	0.120	0.113	0.164	0.126	0.108	0.105	0.110
**Sex**	0.090	0.095	0.098	0.109	0.102	0.099	0.107	0.112	0.106	0.103	0.102	0.073	0.103	0.106	0.103	0.103	0.102	0.153	0.117	0.103	0.090	0.100
**PSW**	0.105	0.104	0.104	0.115	0.108	0.110	0.115	0.121	0.114	0.108	0.120	0.115	0.084	0.109	0.110	0.113	0.115	0.167	0.145	0.112	0.113	0.108
**GO**	0.108	0.128	0.117	0.117	0.114	0.117	0.121	0.122	0.119	0.121	0.126	0.113	0.129	0.089	0.123	0.121	0.137	0.173	0.159	0.115	0.110	0.113
**ET**	0.094	0.095	0.108	0.104	0.105	0.109	0.115	0.113	0.109	0.108	0.125	0.120	0.112	0.118	0.078	0.112	0.118	0.156	0.122	0.107	0.106	0.105
**UD**	0.118	0.118	0.131	0.111	0.113	0.115	0.122	0.123	0.121	0.117	0.137	0.128	0.126	0.128	0.119	0.090	0.122	0.177	0.153	0.119	0.114	0.123
**RLS**	0.101	0.104	0.100	0.097	0.089	0.099	0.096	0.112	0.107	0.096	0.104	0.105	0.101	0.104	0.099	0.107	0.075	0.148	0.123	0.098	0.098	0.102
**LPA**	0.126	0.146	0.139	0.137	0.145	0.155	0.152	0.151	0.155	0.157	0.151	0.138	0.153	0.156	0.149	0.155	0.154	0.151	0.156	0.132	0.127	0.130
**IDS**	0.126	0.114	0.106	0.114	0.111	0.118	0.121	0.137	0.120	0.132	0.117	0.120	0.119	0.119	0.122	0.120	0.122	0.168	0.101	0.118	0.111	0.109
**MS**	0.101	0.101	0.105	0.108	0.103	0.110	0.112	0.112	0.111	0.106	0.109	0.112	0.111	0.111	0.115	0.110	0.113	0.176	0.122	0.074	0.106	0.106
**FR**	0.098	0.099	0.105	0.099	0.103	0.111	0.107	0.113	0.109	0.108	0.114	0.109	0.110	0.103	0.108	0.106	0.103	0.151	0.129	0.104	0.071	0.108
**IL**	0.097	0.104	0.107	0.107	0.100	0.118	0.104	0.113	0.112	0.110	0.132	0.107	0.115	0.118	0.114	0.115	0.113	0.161	0.132	0.107	0.108	0.077

**Table 5:** The DEMATEL technique for ranking of defined factors.

Criteria and Extracted Factors	$\left(R_{i}\right)$	$\left(C_{i}\right)$	${\mathrm{im}}_{i}\;=\;\left(r_{i}\;+\;c_{i}\right)$	${\mathrm{ef}}_{i}\;=\;\left(r_{i}\;-\;c_{i}\right)$
Education (ED)	2.336	2.247	4.583	0.089
Neuroendocrine conditions (NC)	2.221	2.263	4.485	−0.042
Menopause (ME)	2.371	2.277	4.648	0.093
Prenatal determinants (PD)	2.362	2.342	4.704	0.020
Surrounding society and culture (SSC)	2.374	2.268	4.642	0.106
Economic (EC)	2.365	2.386	4.751	−0.021
Marketing (MA)	2.336	2.458	4.795	−0.122
Obesogenic environment (OE)	2.423	2.521	4.944	−0.097
Family influences (FI)	2.427	2.452	4.880	−0.025
Peer influences (PI)	2.430	2.405	4.835	0.026
Age	2.456	2.484	4.940	−0.028
Sex	2.276	2.416	4.692	−0.139
Perceived and stress of work (PSW)	2.517	2.518	5.034	−0.001
Genetics of obesity (GO)	2.691	2.467	5.158	0.224
Ethnicity (ET)	2.442	2.399	4.841	0.043
Unhealthy Diet (UD)	2.726	2.482	5.209	0.244
Relative Living Status (RLS)	2.265	2.497	4.762	−0.231
Lack of physical activities (LPA)	3.212	3.518	6.730	−0.307
Insufficient or deficient sleep (IDS)	2.644	2.883	5.528	−0.239
Marital Status (MS)	2.433	2.291	4.724	0.142
Family Reasons(FR)	2.368	2.236	4.605	0.132
Irregular life (IL)	2.470	2.338	4.808	0.132

**Table 6:** Extracted fuzzy rules.

Rule #	LPA	IDS	GO	PSW	UD	Adult Obesity
1	High	High	High	High	High	Very-High
2	Medium	Low	Medium	Medium	Medium	High
3	Low	Medium	High	High	Low	Medium
4	High	Low	Low	Low	Low	Medium
5	Low	Medium	Low	Low	Low	Low
	⋮	⋮	⋮	⋮	⋮	⋮
24	Medium	Low	Medium	Low	Low	Low
25	Low	High	High	Low	High	Medium

## Data Availability

The analyzed dataset has been included as Supplementary Materials.

## Supplementary Materials

The following supporting information can be downloaded at: https://www.mdpi.com/article/10.3390/ijerph192315432/s1.

## Abbreviations

The following abbreviations are used in this manuscript:

BMI	Body Mass Index
MCDM	Multi Criteria Decision Making
DEMATEL	Decision Making Trial and Evaluation Laboratory
MFRB	Mamdani Fuzzy Rule-Based System
SEM	Structural Equation Modeling
ED	Education
NC	Neuroendocrine Conditions
ME	Menopause
PD	Prenatal Determinants
SSC	Surrounding Society and Culture
EC	Economic
MA	Marketing
OE	Obesogenic Environment
FI	Family Influences
PI	Peer Influences
PSW	Perceived and Stress of Work
GO	Genetics of Obesity
ET	Ethnicity
UD	Unhealthy Diet
RLS	Relative Living Status
LPA	Lack of Physical Activities
IDS	Insufficient or Deficient Sleep
MS	Marital Status
FR	Family Reasons
IL	Irregular Life
BWM	Best Worst Method
AHP	Analytical Hierarchy Process

## References

[1] Cárceles C.M., Fernández-Varón E., Marín P., Escudero E. (2007). Tissue disposition of azithromycin after intravenous and intramuscular administration to rabbits. Vet. J..

[2] Lovrenovic Z., Doumit M. (2019). Development and testing of a passive Walking Assist Exoskeleton. Biocybern. Biomed. Eng..

[3] (2012). Obesity and Overweight. SpringerReference.

[4] Ariaratnam S., Rodzlan Hasani W.S., Krishnapillai A.D., Abd Hamid H.A., Jane Ling M.Y., Ho B.K., Ghazali S.S., Tohit N.M., Mohd Yusoff M.F. (2020). Prevalence of obesity and its associated risk factors among the elderly in Malaysia: Findings from the National Health and Morbidity Survey (NHMS) 2015. PLoS ONE.

[5] Young N., Atan I.K., Rojas R.G., Dietz H.P. (2018). Obesity: How much does it matter for female pelvic organ prolapse?. Int. Urogynecology J..

[6] Lee Y.Y., Muda W.A.M.W. (2019). Dietary intakes and obesity of malaysian adults. Nutr. Res. Pract..

[7] Maziah M., Saemah R., Nooraziah J. (2015). Child-friendly Approches: Choosing the Best Educational Psychology Tool to Teach Healthy Behaviour for Kids. Procedia Soc. Behav. Sci..

[8] Kasirye F., Wahid N. (2020). Factors Influencing Obesity among Malaysian Young Adults in Kuala Lumpur. Asian J. Res. Educ. Soc. Sci..

[9] Abdul Kadir A.B., Abdul Aiman A.G. (2013). National Health and Morbidity Survey 2015. arXiv.

[10] Gopalakrishnan S., Ganeshkumar P., Prakash M.V.S., Christopher A.V. (2012). Prevalence of overweight/obesity among the medical students, Malaysia. Med. J. Malays..

[11] Jan Mohamed H.J.B., Yap R.W.K., Loy S.L., Norris S.A., Biesma R., Aagaard-Hansen J. (2015). Prevalence and determinants of overweight, obesity, and type 2 diabetes mellitus in adults in Malaysia. Asia-Pac. J. Public Health.

[12] Mahaletchumy A., Rampal L., Sharif Z.M. (2019). Prevalence of overweight/obesity and its associated factors among secondary school students in semi urban area in Malaysia. Med. J. Malays..

[13] Peng F.L., Hamzah H.Z., Nor N.M., Saidm R. (2018). Burden of disease attributable to overweight and obesity in Malaysia. Malays. J. Public Health Med..

[14] Kou G., Lu Y., Peng Y., Shi Y. (2012). Evaluation of classification algorithms using MCDM and rank correlation. Int. J. Inf. Technol. Decis. Mak..

[15] Asadi S., Nilashi M., Abumalloh R.A., Samad S., Ahani A., Ghabban F., Yusuf S.Y.M., Supriyanto E. (2022). Evaluation of Factors to Respond to the COVID-19 Pandemic Using DEMATEL and Fuzzy Rule-Based Techniques. Int. J. Fuzzy Syst..

[16] Rao S.H. (2021). A hybrid MCDM model based on DEMATEL and ANP for improving the measurement of corporate sustainability indicators: A study of Taiwan High Speed Rail. Res. Transp. Bus. Manag..

[17] Ghag N., Acharya P., Khanapuri V. (2022). Prioritizing the Challenges Faced in Achieving International Competitiveness by Export-Oriented Indian SMEs: A DEMATEL Approach. Int. J. Glob. Bus. Compet..

[18] Lin W.R., Wang Y.H., Hung Y.M. (2020). Analyzing the factors influencing adoption intention of internet banking: Applying DEMATEL-ANP-SEM approach. PLoS ONE.

[19] Zhao G., Irfan Ahmed R., Ahmad N., Yan C., Usmani M.S. (2021). Prioritizing critical success factors for sustainable energy sector in China: A DEMATEL approach. Energy Strategy Rev..

[20] Si S.L., You X.Y., Liu H.C., Zhang P. (2018). DEMATEL Technique: A Systematic Review of the State-of-the-Art Literature on Methodologies and Applications. Math. Probl. Eng..

[21] Demirci S.E., Canımoğlu R., Elçiçek H. (2022). Analysis of causal relations of marine accidents during ship navigation under pilotage: A DEMATEL approach. Proc. Inst. Mech. Eng. Part J. Eng. Marit. Environ..

[22] Susilowati D. (2011). The Relationship Between Overweight and Socio Demographic Status Among Adolescent Girls in Indonesia. Bul. Penelit. Sist. Kesehat..

[23] Symons C., Polman R., Moore M., Borkoles E., Eime R., Harvey J., Craike M., Banting L., Payne W. (2013). The relationship between body image, physical activity, perceived health, and behavioural regulation among year 7 and year 11 girls from metropolitan and rural Australia. Ann. Leis. Res..

[24] Mubarak A.G.A., Rajan R., Malapan K., Vasudeavan V.K., Mahmood N.R.K.N., Gee T., Ahmad H. (2021). Patient and procedure selection for bariatric and metabolic surgery in Malaysia- the Malaysian consensus. Med. J. Malays..

[25] Sari K., Mansyur M. (2012). Female, live in urban, and the existence of a caregiver increased risk overnutrition in elderly: An Indonesian national study 2010. Health Sci. J. Indones..

[26] Omari R. (2014). Fast Food in Ghana’s Restaurants: Prevalence, Characteristics and Relevance. An Interdisciplinary Perspective. Doctoral Dissertation.

[27] Roemling C., Qaim M. (2012). Obesity trends and determinants in Indonesia. Appetite.

[28] El Rhazi K., Nejjari C., Zidouh A., Bakkali R., Berraho M., Gateau P.B. (2011). Prevalence of obesity and associated sociodemographic and lifestyle factors in Morocco. Public Health Nutr..

[29] Hu F.B. (2009). Social Determinants of Obesity.

[30] Wang Y.C., McPherson K., Marsh T., Gortmaker S.L., Brown M. (2011). Health and economic burden of the projected obesity trends in the USA and the UK. Lancet.

[31] Katikireddi S.V., Skivington K., Leyland A.H., Hunt K., Mercer S.W. (2017). The contribution of risk factors to socioeconomic inequalities in multimorbidity across the lifecourse: A longitudinal analysis of the twenty-07 cohort. BMC Med..

[32] Guedes D.P., Rocha G.D., Silva A.J.R.M., Carvalhal I.M., Coelho E.M. (2011). Effects of social and environmental determinants on overweight and obesity among Brazilian schoolchildren from a developing region. Rev. Panam. De Salud Pública.

[33] Payne-Sturges D., Gee G.C., Crowder K., Hurley B.J., Lee C., Morello-Frosch R., Rosenbaum A., Schulz A., Wells C., Woodruff T. (2006). Workshop Summary: Connecting social and environmental factors to measure and track environmental health disparities. Environ. Res..

[34] Golla V., Lartey G.K., Khubchandani J., Watkins C.M., Student D. (2008). Worker’S Perception: Environmental Factors Influencing Obesity At the Workplace. Am. J. Health Stud..

[35] Syrkiewicz-Świtała M., Detyna B., Sosada N., Detyna J., Świtała R., Bitkowska A., Szkutnik J. (2021). Mobile applications and eating habits among women and men – Polish experiences. Biocybern. Biomed. Eng..

[36] Harrison C.L., Teede H.J., Kozica S., Zoungas S., Lombard C.B. (2017). Individual, social and environmental factors and their association with weight in rural-dwelling women. Aust. N. Z. J. Public Health.

[37] Reuter C.P., De Mello E.D., Da Silva P.T., Borges T.S., Klinger E.I., Franke S.I.R., Valim A.R.M. (2018). Overweight and Obesity in Schoolchildren: Hierarchical Analysis of Associated Demographic, Behavioral, and Biological Factors. J. Obes..

[38] Safaei M., Sundararajan E.A., Driss M., Boulila W., Shapi’i A. (2021). A systematic literature review on obesity: Understanding the causes & consequences of obesity and reviewing various machine learning approaches used to predict obesity. Comput. Biol. Med..

[39] Ghosh S., Bouchard C. (2017). Convergence between biological, behavioural and genetic determinants of obesity. Nat. Rev. Genet..

[40] Sweeting H.N. (2008). Gendered dimensions of obesity in childhood and adolescence. Nutr. J..

[41] Asadi S., Nilashi M., Iranmanesh M., Hyun S.S., Rezvani A. (2021). Effect of internet of things on manufacturing performance: A hybrid multi-criteria decision-making and neuro-fuzzy approach. Technovation.

[42] Costa F., Denis Granja A., Fregola A., Picchi F., Portioli Staudacher A. (2019). Understanding Relative Importance of Barriers to Improving the Customer–Supplier Relationship within Construction Supply Chains Using DEMATEL Technique. J. Manag. Eng..

[43] Shevyakova A., Munsh E., Arystan M., Petrenko Y. (2021). Competence development for Industry 4.0: Qualification requirements and solutions. Insights Reg. Dev..

[44] Peleckis K. (2021). Application of the dematel model for assessing it sector’s sustainability. Sustainability.

[45] De S.K. (2020). On Degree of Fuzziness and Fuzzy Decision Making. Cybern. Syst..

[46] Dimitriou L., Tsekeris T., Stathopoulos A. (2008). Adaptive hybrid fuzzy rule-based system approach for modeling and predicting urban traffic flow. Transp. Res. Part C Emerg. Technol..

[47] Liu H., Zhang L. (2018). Fuzzy rule-based systems for recognition-intensive classification in granular computing context. Granul. Comput..

[48] Mamdani E.H. (1974). Application of Fuzzy Algorithms for Control of Simple Dynamic Plant. Proc. Inst. Electr. Eng..

[49] Pandey D.C., Kushwaha G.S., Kumar S. (2020). Mamdani fuzzy rule-based models for psychological research. SN Appl. Sci..

[50] Tafuro A., Dammacco G., Esposito P., Mastroleo G. (2022). Rethinking performance measurement models using a fuzzy logic system approach: A performative exploration on ownership in waste management. Socio-Econ. Plan. Sci..

[51] Asadi S., Nilashi M., Iranmanesh M., Ghobakhloo M., Samad S., Alghamdi A., Almulihi A., Mohd S. (2022). Drivers and barriers of electric vehicle usage in Malaysia: A DEMATEL approach. Resour. Conserv. Recycl..

[52] Taylor D. (2014). Physical activity is medicine for older adults. Postgrad. Med. J..

[53] Kaur J., Kaur G., Ho B.K., Yao W.K., Salleh M., Lim K.H. (2015). Predictors of physical inactivity among elderly Malaysians: Recommendations for policy planning. Asia-Pac. J. Public Health.

[54] Markwald R.R., Melanson E.L., Smith M.R., Higgins J., Perreault L., Eckel R.H., Wright K.P. (2013). Impact of insufficient sleep on total daily energy expenditure, food intake, and weight gain. Proc. Natl. Acad. Sci. USA.

[55] Beccuti G., Pannain S. (2011). Sleep and obesity. Curr. Opin. Clin. Nutr. Metab. Care.

[56] Buxton O.M., Marcelli E. (2010). Short and long sleep are positively associated with obesity, diabetes, hypertension, and cardiovascular disease among adults in the United States. Soc. Sci. Med..

[57] Gómez-Pérez D., Cancino M., Moreno P.I., Ortiz M.S. (2021). Weight Stigma, Chronic Stress, Unhealthy Diet, and Obesity in Chilean Adults. Int. J. Behav. Med..

[58] Kjøllesdal M.R., Holmboe-Ottesen G., Wandel M. (2011). Frequent use of staff canteens is associated with unhealthy dietary habits and obesity in a Norwegian adult population. Public Health Nutr..

[59] Musaiger A.O., Al-Khalifa F., Al-Mannai M. (2016). Obesity, unhealthy dietary habits and sedentary behaviors among university students in Sudan: Growing risks for chronic diseases in a poor country. Environ. Health Prev. Med..

[60] Durazzo A., Lemamsha H., Randhawa G., Papadopoulos C. (2022). Investigating the Association between Unhealthy Dietary Habits and Obesity among Libyan Adults. Int. J. Environ. Res. Public Health.

[61] Cureau F.V., Sparrenberger K., Bloch K.V., Ekelund U., Schaan B.D. (2018). Associations of multiple unhealthy lifestyle behaviors with overweight/obesity and abdominal obesity among Brazilian adolescents: A country-wide survey. Nutr. Metab. Cardiovasc. Dis..

[62] Silventoinen K., Jelenkovic A., Sund R., Hur Y.M., Yokoyama Y., Honda C., Hjelmborg J.B., Moller S., Ooki S., Aaltonen S. (2016). Genetic and environmental effects on body mass index from infancy to the onset of adulthood: An individual-based pooled analysis of 45 twin cohorts participating in the COllaborative project of Development of Anthropometrical measures in Twins (CODATwins). Am. J. Clin. Nutr..

[63] Silventoinen K., Rokholm B., Kaprio J., Sørensen T.I. (2010). The genetic and environmental influences on childhood obesity: A systematic review of twin and adoption studies. Int. J. Obes..

[64] Rohde K., Keller M., la Cour Poulsen L., Blüher M., Kovacs P., Böttcher Y. (2019). Genetics and epigenetics in obesity. Metab. Clin. Exp..

[65] Barrington W.E., Beresford S.A., McGregor B.A., White E. (2014). Perceived Stress and Eating Behaviors by Sex, Obesity Status, and Stress Vulnerability: Findings from the Vitamins and Lifestyle (VITAL) Study. J. Acad. Nutr. Diet..

